# Does the safe childbirth checklist (SCC) program save newborn lives? Evidence from a realistic quasi-experimental study, Rajasthan, India

**DOI:** 10.1186/s40748-019-0098-4

**Published:** 2019-03-01

**Authors:** Beena Varghese, Andrew Copas, Shwetanjali Kumari, Souvik Bandyopadhyay, Jigyasa Sharma, Somen Saha, Vikas Yadav, Somesh Kumar

**Affiliations:** 1 Public Health Foundation of India, Plot No 47, Sector 44, Gurugram, Haryana 122002 India; 2 University College, London, UK; 3 Independent Public health Consultant (Previously with PHFI), Banaglore, India; 4 Harvard T. H Chan School of Public Health, Boston, USA; 5 Jhpeigo, Okhla Industrial Estate Phase 3 Rd, New Delhi, Delhi, 110020 India

**Keywords:** Safe childbirth checklist, Maternal and newborn care, Stillbirths, Very early neonatal mortality, Facility-based maternal and newborn interventions

## Abstract

**Background:**

The WHO Safe Childbirth Checklist (SCC) is a facility-based reminder tool focusing on essential care to improve quality of intrapartum care. We aimed to assess the impact of an intervention package using the SCC tool on facility-based stillbirths (SBs) and very early neonatal deaths (vENDs), in Rajasthan, India.

**Methods:**

Within a quasi-experimental framework, districts were selected as intervention or comparison, matched by annual delivery load. The SCC tool was introduced at all district and sub-district level health facilities in the seven intervention districts, followed by monthly supportive supervision visits. In addition, supply of drugs and equipment were facilitated in all facilities (2013–2015). Facilities in the comparison districts provided routine care. Analysis included only the facilities with a specialized newborn care unit and information on all births was collected from facility registers. The primary outcome was the combined facility-based stillbirths and very early neonatal deaths (within 3-days after birth). We used generalized estimating equation with a Poisson regression model, with time as a linear term and adjusted for facility type in our model to estimate the effect of the intervention. [ClinicalTrials.gov: NCT01994304].

**Results:**

77,239 births were recorded from 19 intervention facilities and 59,800 births from 15 comparison facilities. The intervention facilities reported 1621 stillbirths and 505 vENDs compared to 1390 stillbirths and 420 vENDs from the comparison facilities (RR 0.89, CI 0.81, 0.97). This translated to 11.16% (*p* = 0.01) reduction in total mortality (11.39% in stillbirths alone) in the intervention facilities.

**Conclusion:**

Our results suggest that the SCC program is an effective intervention that could potentially avert 40,000 intrapartum deaths in India annually, most of reduction coming from prevention of stillbirths.

## Introduction

Globally an estimated 2.7 million neonatal deaths and an additional 2.6 million stillbirths occur annually [1–3]. About 70% of neonatal deaths reported are within the first week, and 36% on the day of birth [4]. Achieving the Sustainable Development Goal (SDG 3∙2) of reducing global neonatal mortality to 12 per 1000 live births therefore demands a significant focus on improving quality of care during childbirth to reduce early neonatal deaths [5–7]. India carries the highest share of global stillbirths (23%) and neonatal mortality (26%) in 2015 [1–3]. As a measure to reduce preventable mortality and morbidity, India successfully increased the proportion of facility-based births, primarily through its conditional cash transfer program, however, early evaluations indicate that these efforts have a modest impact on reducing mortality [8, 9]. This has been primarily attributed to inadequate attention to quality of both routine and emergency obstetric and newborn care [10, 11]. Currently 83% of births in India are facility-based births. This has well positioned India to benefit from cost-effective interventions that can improve quality of facility-based childbirth care and avert preventable deaths [12, 13].

The WHO Safe Childbirth Checklist (SCC) is a facility-based reminder tool aimed to assist healthcare workers in improving maternal and newborn care practices, before, during, and after delivery, thereby expected to impact on perinatal mortality [14]. A pilot study of the WHO SCC tool in a sub-district facility in southern India indicated marked improvement in the delivery of essential maternal and newborn care practices [15]. Another study in a tertiary centre in Sri Lanka reported that the SCC tool was acceptable to healthcare workers [16]. Other observational studies from district and sub-district level facilities in Rajasthan, India; Gobabis District Hospital from Namibia; and Masaka District Hospital from Rwanda where WHO SCC tool were used as part of a quality improvement initiative, reported significant increases in SCC targeted essential maternal and newborn care practices [17–19]. The recent cluster-randomized trial of SCC intervention in northern India (Better-Birth trial) where WHO SCC tool was used with peer coaching (at sub-district and primary health care facilities) also reported improved uptake of and provider adherence to essential birth practices (EBPs) [20]. However, this study did not find any impact of SCC on perinatal death, maternal death, or maternal severe complications within 7 days after delivery [21].

For our study, the SCC program was implemented only at district and sub-district level facilities in the state of Rajasthan and we used a pragmatic mixed-methods design to study the feasibility, effectiveness and cost-effectiveness of the WHO SCC-based program in preventing intrapartum mortality [22]. In this paper, we report findings on the effectiveness of the SCC program in reducing facility-based stillbirths (SBs) and very early neonatal deaths (vENDs, deaths within three-days after birth).

## SCC program in Rajasthan

The Government of Rajasthan, India, with technical support from Jhpiego (a John Hopkins University-affiliate, international, non-profit health organization) implemented the SCC program at district hospitals (DH, 100–500 bed facilities) and sub-district level health facilities (30–50 bed facilities) that provide secondary level care across select districts in Rajasthan, India. To select the intervention and comparison districts, we used annual delivery load of the district and sub-district level facilities, (from State Pregnancy and Child Tracking System, PCTS), Neonatal mortality rate (Annual health survey 2012–2013), data on demographic indicators, and the operational feasibility for doing this study in the district (Tables 1 and 2). Based on these, we selected seven intervention districts and six comparison districts (with 100 facilities in each group) for the implementation of the SCC program.. We also used socio-demographic (from Census 2011), and, stillbirth rate, (PCTS 2011–2012) along with indicators from a rapid assessment survey by Jhpiego (Table 1). This survey provided information on human resources, infrastructure and supplies for all intervention and comparison facilities (Tables 1 and 2). The government was informed of all 13 selected study districts, which ensured that no other major maternal and newborn health interventions were introduced in these districts during the study period (2013 to 2015).

**Table 1:** Comparison of Intervention and Comparison Districts for Safe Childbirth Checklist Intervention, at Baseline (2011–12)

Indicators	Intervention Districts	Comparison districts	Source
Socio-Demographic indicators			Census 2011
Population (total)	2,148,061	2,507,706	
Literacy rate (%)	63	70	
Proportion Households having Monthly income Less than Rs. 5000)	67	63	
Maternal & child health indicators			Annual Survey, 2012–2013
Institutional deliveries (%) Rural	77	79	
Mothers who received any Antenatal check-up (%)	49	52	
Children breastfed within one hour of birth (%)	55	63	
NMR Rural (per 1000 live births)	39∙67	40.71∙	
Pregnancy and Child Tracking System (PCTS,)			PCTS 2011–2012
Delivery load (Annual)	120,480	108,887	
SBR per 1000 births (for 34 study facilities)^a^	23∙45	24∙10	
Rapid assessment survey by Jhpiego (district data)			2012
Infrastructure (%)			
Electricity backup	88	89	
Running water for hand-washing	87	89	
Availability of blood bank and blood storage	15	21	
Human Resource (compared against Indian Public Health Standard %)			
At District Hospitals			
Specialists (Obstetrician/Gynecologist)	100	93	
Pediatrician	112	93	
Staff nurses	30	43	
At Community Health Centres			
Specialists (Obstetrician/Gynecologist)	15	31	
Pediatrician	30	33	
Staff nurses	59	52	
Staff nurses and ANMs trained in skill birth attendant (SBA)	19	20	
Availability of Oxytocin	97	95	
Magnesium sulphate	18	20	

^a^Stillbirths for one sub-district facility is not recorded in system due to some acknowledged technical issues, thus was extrapolated using stillbirth rates for that facility from our data

**Table 2:** List of Intervention and Comparison Districts with Delivery load and Neonatal Mortality Rate (NMR) in 2011–12

Districts	Deliveries	NMR	Districts	Deliveries	NMR
Intervention	CHC/SDH/DH	per 1000	Comparison	CHC/SDH/DH	per 1000
Alwar	39,376	35	Bharatpur	32,451	42
Jhalore	9094	58	Pali	18,075	41
Sirohi	8622	41			
Bikaner	18,924	37	Jodhpur	18,924	37
Dausa	12,726	33	Jaipur II	12,692	37
Churu	14,856	36	Jhunjhunu	8427	39
Sikar	16,882	45	Nagaur	18,318	42
Average	17,211	40.71		18,148	39.67
Total	120,480			108,887	

*CHC* Community Health Centre, *SDH* Sub-District Hospital, *DH* District Hospital

In Rajasthan, the WHO SCC tool was integrated into the client case-sheet, designed to act as a job-aid and a reminder tool, aiming to improve adherence to evidence-based practices for childbirth and newborn care as well as act as an accountability tool as it contained signature of the provider at each pause point/critical points in the delivery. The SCC program or intervention (2013 to 2015), implemented across the seven intervention districts (100 facilities), included (1) one and a half day orientation of the modified SCC tool to nurses and medical officers attending the labor room at each facility, (2) the introduction of the SCC tool in the labor rooms of all district hospitals and community health centres (CHCs--sub-district level health facilities), and (3) fortnightly to monthly supportive supervision visits to provide on-site support to the providers in using the SCC and for monitoring adherence to EBPs. In the comparison districts, routine care provision continued at all facilities. Supply of essential supplies and drugs through the government procurement system was facilitated by the implementing agency in both intervention and comparison districts, to avoid any potential bias. All efforts were made to ensure that the incremental inputs for implementation of this checklist was minimal, quantified, scalable and sustainable.

## Evaluation methods

### Study design, data sources and outcome

**Fig. 1:**
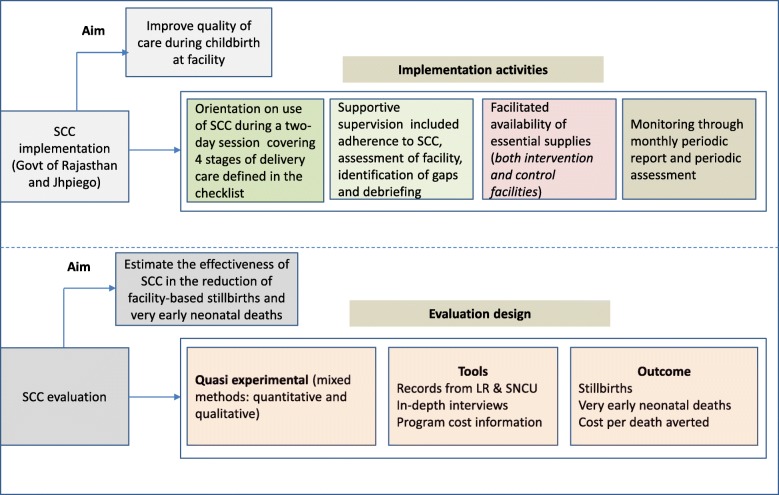
Safe Childbirth Checklist implementation and evaluation framework

**Fig. 2:**
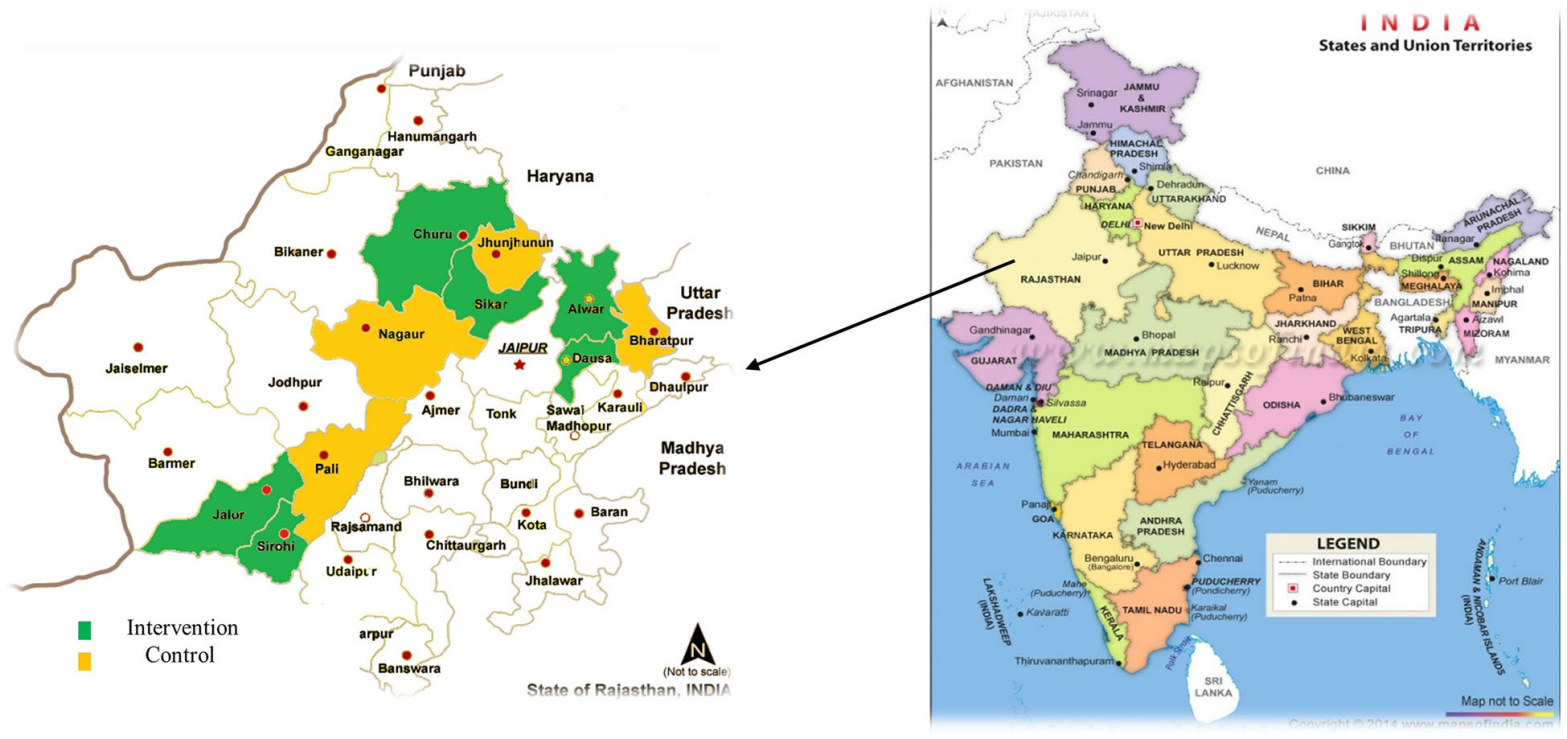
Safe Childbirth Checklist Evaluation Study Districts (6 intervention and 4 comparison districts)

We used a mixed-methods (quantitative and qualitative) approach within a quasi-experimental post-only cluster design to evaluate the SCC program (Fig. 1). For evaluation, we included only the facilities with special newborn care units (SNCUs)--SNCUs provide care to sick newborns^1^ and are located adjacent to labor rooms at all DH and at large CHCs. They are the primary source of information for facility-based very early neonatal deaths, while labor rooms recorded all stillbirths. There were 19 intervention facilities with SNCUs (six were DH) across six districts and 15 comparison facilities with SNCUs (4 DH) across 4 districts (Fig. 2).

Data on all deliveries were collected from facility registers in the labor rooms and in the SNCUs between November 2013 and April 2015 in a phased manner, in alignment with the phased implementation design. To ensure that sufficient time was given for the SCC program to be institutionalized, there was at least a six-month interval between the initial SCC orientation of the providers and the data collection for evaluation. Every facility provided 14 months of birth and outcome data. In the SNCUs, newborn with severe complications were further referred out to tertiary care centres. Those referred out within three days of birth were tracked through phone calls to estimate the mortality rate among such referred cases. Strict quality control including validation of all recorded deaths was maintained throughout the data collection process. Methodological details related to electronic data collection, data management and quality control is described elsewhere [22].

The primary outcome of the study is a combined metric of facility-based stillbirths and vENDs. For our study, we defined stillbirth as late foetal death occurring at or beyond 28 weeks of gestation or with a birth-weight of at least 1000 g [23, 24]. We used gestational age (as recorded in the facility registers) as the primary criteria for classifying stillbirths. For those cases, where gestational age was missing (*N* = 495), we used the recorded birth-weight information. Stillbirth included both macerated and fresh stillbirths, as such level of distinction was not available in the records. Facility-based vENDs is defined as a newborn death within three days after birth. This was calculated using dates of birth and death recorded in the registers and was mainly obtained from SNCUs.

### Sample size

We aimed to detect a 15% reduction in our composite outcome (stillbirths and vENDs) assuming base mortality rate of 30/1000 births. Ignoring clustering at the district level, which we assumed to be minimal, we accounted for clustering at the facility level by assuming an intra-cluster correlation (ICC) of 0∙0002, slightly higher than the ICC for stillbirths used in a similar study in India [25]. We estimated an average facility cluster size of 3183 births per year and a coefficient of variation in cluster size of 0∙849 (from previous year data). The design effect was estimated at 2∙043 [26]. We aimed to collect data from 19 intervention facilities and 15 comparison facilities for at least a year, giving a sample size of at least 60,477 births in the intervention arm and 47,745 in the comparison arm. Using 5% level of significance and acknowledging the allocation ratio 19:15, the power to detect the targeted reduction is estimated to be 88%.

### Statistical analysis

Our primary analysis assessed the relationship between the SCC program and newborn outcomes. We used generalized estimating equation with a Poisson regression model to estimate the effect of the SCC program on stillbirths and vENDs. Robust standard errors were computed to account for facility-level clustering with an exchangeable correlation structure. To address potential confounding, we assessed the relationship between treatment status and covariates like gestational age, birth-weight, maternal age, place of delivery (DH or CHC), type of delivery (vaginal or caesarean section), newborn sex, parity, and birth type (singleton or multiple birth). In studies with large sample sizes, trivial differences may attain statistical significance [27, 28], therefore, we adjusted for covariates with clinically meaningful differences between intervention and comparison.

We included time as a linear term and adjusted for facility type in our model. The interaction between facility type and time was not significant and hence not included in the model. We also conducted a sub-analysis by facility type and found that interaction was not significant and thus was not included in this paper. We included the logarithm of the total births at a facility as an offset in the model, a standard technique to model death rate. This model allowed us to estimate the change in mortality rate associated with the SCC program.

Confidence intervals for estimates of the death rate were obtained following the procedure described in Clopper and Pearson [29]. All statistical analysis was done using R 3∙1∙2 [30]. Data collection and analysis were conducted by independent researchers who were blinded to the type of facility (intervention or comparison).

This study was approved by the Institutional Ethics Committee of the Public Health Foundation of India (TRC-IEC-141/12) and by the Government of Rajasthan. The study is registered at the Clinical Trials website of the U.S. Government, ClinicalTrials.gov: NCT01994304.

## Results

**Fig. 3:**
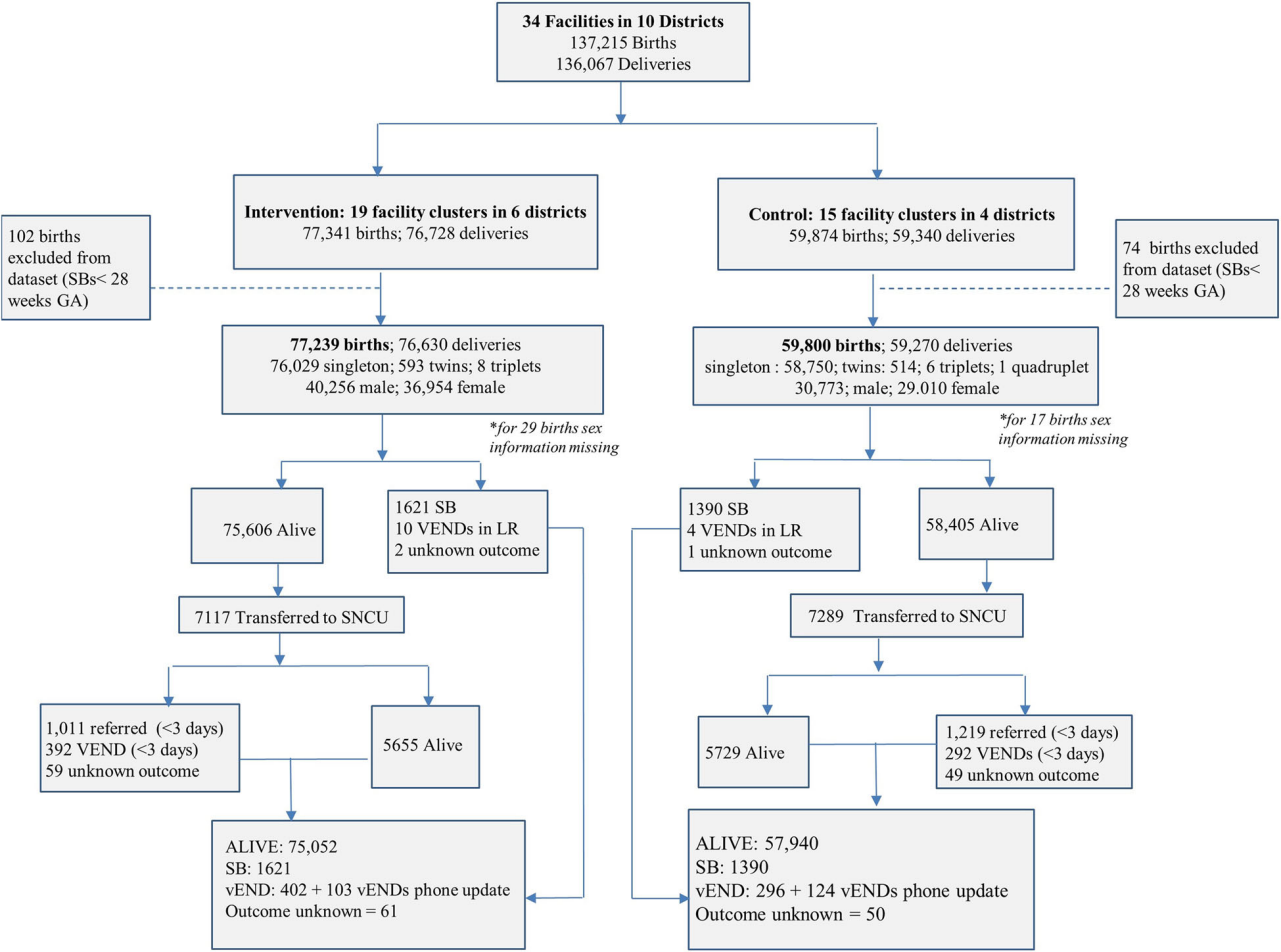
Evaluation study profile

The study included 137,215 births from the 19 intervention and 15 comparison facilities. After excluding 176 births with reported gestational age less than 28 weeks, we had a total of 137,039 facility-based births, of which 56% were in intervention facilities (Fig. 3).

About 98% of births were singleton, 52% male in both intervention and comparison facilities. Labor rooms in the intervention facilities reported 1621 stillbirths and 10 vENDs, and the remainder 75,606 were alive. The comparison facilities recorded 1390 stillbirths and 4 vENDs, and 58,405 live births. Of these live cases, 9% (7117) cases in intervention and 12% (7289) in comparison facilities were transferred to SNCUs. Among these, 392 vENDs and 1011 referrals from intervention compared to 292 vENDs and 1219 referrals from comparison facilities were reported. Phone tracking of these referred cases yielded 103 vENDs from intervention and 124 vENDs from the comparison groups. In total, there were 75,052 live births and 2126 deaths (SBs and vENDs) in intervention group and 57,940 live cases and 1810 deaths in comparison group (Fig. 3).

Table 3 shows maternal and neonatal characteristics.. For the study population, mean maternal age was 24∙4 ± 3∙5 years, gestational age was 37∙6 ± 1∙3 weeks and birth weight was 2∙8 ± 0∙5kgs. Almost all obstetric and demographic covariates were comparable between control and intervention facilities. Although the differences for maternal age, parity, and birth-weight were statistically significant at 1% level (Table 3), a function of large sample size, the differences were inconsequential (birth-weight intervention 2∙76 kg, comparison 2∙77 kg; maternal age 24∙35 vs. 24∙43 years; Nulliparous 31.89% vs. 32.33%). Facility type showed a significant difference with 57% in intervention group with birth at district hospital compared to 53% of births in a DH for the comparison group.

**Table 3:** Maternal and newborn characteristics of study population

Category	Intervention	Comparison	*P* values
Total Births	77,239 (56%)	59,800 (44%)	< 0∙001
Facility Type			< 0∙001
District Hospital	44,224 (57%)	31,532 (53%)	
Maternal mean age in years [SD]	24∙35 (3∙42)	24∙43 (3∙52)	< 0∙001
Gestational mean age in weeks [SD]	37∙54 (1∙48)	37∙54 (1∙11)	0∙066
Type of delivery			0∙976
Vaginal	72,602 (94%)	56,383 (94%)	
Sex			0∙054
Male	40,256 (52%)	30,773 (51%)	
Parity			< 0∙001
Nulliparous	24,634 (31.8%)	19,335 (32.3%)	
Birth weight (Kilograms) [SD]	2∙76 (0∙49)	2∙77 (0∙47)	< 0∙001

Table 4 shows birth and death outcomes along with adjusted (facility type) relative risks and reduction in mortality rate associated with the intervention. The primary analysis showed that the facility-based mortality (stillbirths and vENDs) was 27∙52 per 1000 births in the intervention clusters compared to 30∙26 per 1000 births in the comparison clusters. The adjusted relative risk of the total mortality was estimated to be 0∙89 [95% CI: 0∙81, 0∙97], which translates to a statistically significant 11∙16% [95% CI: 2∙78%, 18∙82%] reduction in combined mortality. Reduction in stillbirths alone was significant at 11.39% [95% CI: 2∙47, 19.5%] (Table 4).

**Table 4:** Impact of Safe Childbirth Checklist program on facility-based stillbirth and very early neonatal deaths

Birth outcome (Facility-Based)	Intervention	Comparison	Adjusted Relative Risk^a^ (95% CI)	Percentage Reduction in Mortality (95% CI)
Total Births	77,239	59,800		
Stillbirths and very early neonatal deaths (less than three days after birth)-Total death	2126	1810		
Total death rate/1000 total births	27∙52	30∙26	0∙89 [0∙81, 0∙97]	11∙16 [2∙78, 18∙82]
Stillbirths	1621	1390		
Stillbirth rate/1000 total births	20∙99	23∙24	0∙89 [0∙81, 0∙98]	11∙39 [2∙47, 19∙50]
Very early neonatal deaths	505	420		
Very early neonatal death rate/1000 live births	6∙73	7∙25	0∙90 [0∙76, 1∙06]	10∙35 [−6∙42, 24∙49]

^a^Adjusted for linear time trend and type of facility (DH and CHC) acility (DH and CHC)

## Discussion

**Fig. 4:**
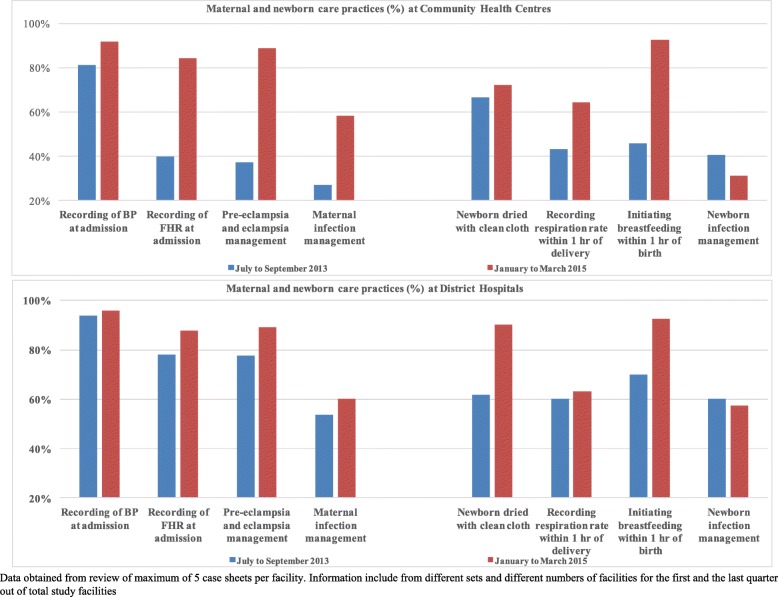
Adherence to Maternal and Newborn care practices at the beginning and end of the SCC intervention at Community Health Centres and District Hospitals (Intervention facilities)

Our evaluation study found that the SCC-based intervention in Rajasthan is associated with a statistically significant reduction of 11∙16% (*p*-value = 0∙01) in stillbirths and very early neonatal deaths, 77% of which was contributed by stillbirths alone. In terms of impact of the intervention at different levels of health facilities, findings from program monitoring showed that changes in provider behavior (between start and end of intervention) at the CHCs (sub-district level) were much higher (average of 64%) than at district level facilities (18%) (Fig. 4). The highest difference was for management of severe preeclampsia/eclampsia and infection management. Furthermore, in our qualitative interviews, nurses at the CHCs also reported that the SCC intervention resulted in early identification, management and timely referral of pregnancy-related complications, mainly for pre-eclampsia [31]. Nurses at the district level facilities reported that they were adhering to many of the practices in the checklist even prior to the intervention; nevertheless, found the checklist to be an useful reminder tool.

A recent observational study related to SCC use from Namibia also reported a significant reduction is perinatal mortality, largely due to drop in fresh stillbirths [18]. An observational study done in parallel using a sample of facilities from the SCC implementation in Rajasthan, India (done by the implementing partner Jhpiego) reported that the adherence to almost all SCC practices especially, pre-eclampsia management, postpartum hemorrhage and infection management were significantly higher in the intervention groups than in the control groups [17]. Many of these improved care practices may reduce incidence of birth asphyxia, and complications due to prematurity, which are some of the main causes of fresh stillbirths and early neonatal deaths [32, 33], supporting our hypothesis of improved care practices due to SCC leading to reduction in mortality.

The Better-Birth study, a randomized control trial in Uttar Pradesh, India, however, found no significant impact of the SCC intervention on perinatal or maternal mortality [21]. It is important to note that although both intervention programs were based on the WHO SCC tool, they differed in various key aspects. First, the Better-Birth study facilities are a combination of sub-district and primary-level facilities, whereas those in our study are secondary-level facilities with very different infrastructure and human resource capacity. Second, in contrast to the Rajasthan SCC program, which had monthly supervision visits by Jhpiego staff along with government staff, the Better-Birth intervention provided supportive supervision through a trained peer “coach” with decreasing intensity [20]. Third, availability of equipment, drugs and supplies were not ensured in the Better-Birth study facilities, unlike that in Rajasthan. Some of these crucial health system level differences may explain the different outcomes of these two studies.

Other recent studies on essential newborn care training and community mobilization also have reported mixed effects on mortality: one showing significant reductions in stillbirths in a multi-country trial with the use of before-and-after design [34]; other using pre/post-intervention with active baseline design showing effect only on early neonatal deaths [35]; another, a combined community and facility-based intervention model using cluster-randomized controlled trial showing large but non-significant impact on perinatal and neonatal mortality [36]. All these interventions had some component focused on improving intrapartum care practices, similar to the focus in the SCC program (like recognition and early management of complications, routine neonatal care, initiation of breathing, resuscitation, and thermoregulation), showing varied results. Pasha et al. highlighted the need for a more holistic approach with improved health care infrastructure along with availability of essential supplies and equipment and skilled manpower towards improving pregnancy outcomes [37].

The SCC program in Rajasthan benefited from working within the government systems at secondary level facilities supported by continuous supervision and consistent availability of drugs and supplies. However, we believe, the effect size in this study is still on the conservative side as the beneficial impacts of many improved practices like infection prevention, breastfeeding, management of maternal complications, etc., could not be captured in the current study outcome. We hypothesize potential reduction in incidence of maternal complications and neonatal sepsis attributable to the SCC program. This needs to be further explored by future studies.

Evidence from this study, however, should be interpreted considering several limitations. First, lack of randomization of the intervention limits our inference. Despite robust design and analytical considerations, the post-only quasi-experimental design is limited in its scope causal claim. Second, because of the small number of districts, we did not consider district-level clustering resulting in narrower confidence intervals. However, the heterogeneity of study districts is likely smaller than heterogeneity between facilities within districts. Therefore, this should not substantially alter our findings. Third, our primary data were obtained from facility registers. Thus, it encompasses limitations of administrative data. We acknowledge potential misclassification of very early neonatal deaths as stillbirths in the study facilities, as reported by other studies too [38, 39]. In addition, we have made no distinction between fresh and macerated stillbirths even though the intervention would only affect fresh stillbirths. Obtaining this level of information from records and registers at the facility was not feasible, as the providers did not make this distinction while recording stillbirths. However, studies from India have reported that fresh stillbirths contribute 50 to 80% of total stillbirths [40–42] and a more recent study reported that 30% of stillbirths were attributable to obstetrics complications and excessive bleeding during delivery [43]. Finally, our study sites were the 34 facilities with SNCUs (contributing to almost 60% of total births from all study facilities), impacting the generalizability of our results across all health facilities. This was done to ensure accuracy in the counts of vENDs as they were only reported in SNCUs. However, given that the SCC had the highest impact on reduction of stillbirths, we believe our conclusions are robust.

## Conclusion

In conclusion, our study findings, using a pragmatic study design, indicate that the Safe Childbirth Checklist intervention is an effective intervention to reduce intrapartum mortality. With an annual birth cohort of 25 million births and institutional delivery rate of 80%, our conservative estimate indicate that the scale-up of SCC program in India could avert around 40,000 stillbirths and very early neonatal deaths. Improving clinical capacity of providers along with improved monitoring and accountability could perhaps further enhance the impact of such an intervention. This is in fact the essence of the national quality improvement program ‘Dakshata,’ a strategic initiative designed by the government of India to strengthen quality of institutional delivery care based on the initial findings of the SCC program [44]. With strategic investment in evidence-based intervention such as the SCC program to improve quality of facility based delivery care, maternal and newborn mortality reduction can perhaps be accelerated in India and beyond India.

## Footnotes

1 Except assisted ventilation and major surgeries

## References

[1] UN inter-agency group for child mortality estimation. Levels and trends in child mortality: report 2015. UNICEF, WHO, World Bank Group, United Nations 2015. http://www.who.int/maternal_child_adolescent/documents/levels_trends_child_mortality_2015/en/. Accessed on 20 Nov 2017.

[2] Ending preventable stillbirths. An executive summary for the Lancet Series. Lancet 2016. 1–8. http://www.thelancet.com/pb/assets/raw/Lancet/stories/series/stillbirths2016-exec-summ.pdf. Accessed on 5 Dec, 2017.

[3] Blencowe H, Cousens S, Jassir FB, Say L, Chou D, Mathers C (2016). National, regional, and worldwide estimates of stillbirth rates in 2015, with trends from 2000: a systematic analysis. Lancet Glob Health.

[4] Oza S, Cousens SN, Lawn JE (2014). Estimation of daily risk of neonatal death, including the day of birth, in 186 countries in 2013: a vital-registration and modelling-based study. Lancet Global Health.

[5] Sustainable Development Goals. UN Web Services Section, Department of Public Information, United Nations. Available at http://www.un.org/sustainabledevelopment/health/. 5 Oct 18.

[6] Lawn JE, Kerber K, Enweronu-laryea C, Massee O (2009). Newborn survival in low resource settings — are we delivering ?. BJOG..

[7] Lawn J, Shibuya K, Stein C (2005). No cry at birth : global estimates of intrapartum stillbirths and intrapartum-related neonatal deaths. Bull World Health Organ.

[8] Powell-Jackson T, Mazumdar S, Mills A (2015). Financial incentives in health: new evidence from India’s Janani Suraksha Yojana. J Health Econ.

[9] Ng M, Misra A, Diwan V, Agnani M, Levin-rector A, De Costa A (2014). An assessment of the impact of the JSY cash transfer program on maternal mortality reduction in Madhya Pradesh. India Global Health Action.

[10] Chaturvedi S, Randive B, Diwan V, De Costa A (2014). Quality of obstetric referral services in India’s JSY cash transfer programme for institutional births: a study from Madhya Pradesh province. PLoS One.

[11] Tunçalp Ӧ, Were W, MacLennan C, Oladapo O, Gülmezoglu A, Bahl R (2015). Quality of care for pregnant women and newborns-the WHO vision. BJOG..

[12] Government of India, a digital India initiative. Trends in Child Delivery at Health Facilities (Institutional Delivery) across India from 2008–09 to 2012–13. https://community.data.gov.in/trends-in-child-delivery-at-health-facilities-institutional-delivery-across-india-from-2008-09-to-2012-13/. Accessed 15 Nov 2017.

[13] Ministry of Health and Family Welfare Government of India. Report & Recommendations of the Seventh Common Review Mission 2013; 1–165. Available at http://nrhm.gov.in/images/pdf/monitoring/crm/7th-crm/report/7th_CRM_Main_Report.pdf. Accessed on 18 Dec 2018.

[14] Safe childbirth checklist programme, An overview. 2013. Available at http://www.who.int/patientsafety/implementation/checklists/background_document.pdf?ua=1. Accessed on 11 Nov 2017.

[15] Spector JM, Agrawal P, Kodkany B, Lipsitz S, Lashoher A, Dziekan G (2012). Improving quality of care for maternal and newborn health: prospective pilot study of the who safe childbirth checklist program. PLoS One.

[16] Patabendige M, Senanayake H (2015). Implementation of the WHO safe childbirth checklist program at a tertiary care setting in Sri Lanka: a developing country experience. BMC Pregnancy Childbirth.

[17] Kumar S, Yadav V, Balasubramaniam S (2016). Effectiveness of the WHO SCC on improving adherence to essential practices during childbirth, in resource constrained settings. BMC Pregnancy and Childbirth.

[18] Kabongo L, Gass J, Kivondo B (2017). Implementing the WHO safe childbirth checklist: lessons learnt on a quality improvement initiative to improve mother and newborn care at Gobabis District hospital. Namibia BMJ Open Qual.

[19] Tuyishime E, Park PH, Rouleau D (2018). Implementing the World Health Organization safe childbirth checklist in a district Hospital in Rwanda: a pre- and post-intervention study. Matern Health Neonatol Perinatol.

[20] Marx Delaney M, Maji P, Kalita T, Kara N, Rana D, Kumar K (2017). Improving adherence to essential birth practices using the WHO safe childbirth checklist with peer coaching: experience from 60 public health facilities in Uttar Pradesh. India Global Health Sci Prac.

[21] Semrau KEA, Hirschhorn LR, Delaney MM, et al. Outcomes of implementing a coaching-based WHO Safe Childbirth Checklist program in India. The New England Journal of Medicine. 2017; 10.1056/NEJMoa1701075. 10.1056/NEJMoa1701075 PMC5672590 29236628

[22] Kumari S, Panicker R, Jayaram A, Dumka N, Sharma S, Singhal S (2016). Evaluation of the safe childbirth checklist program in Rajasthan, India. J Public Health Dev Ctries.

[23] Stillbirths. About definition. http://www.who.int/maternal_child_adolescent/epidemiology/stillbirth/en/. Accessed 11 Nov 2017.

[24] Lawn JE, Blencowe H, Pattinson R, Cousens S, Kumar R, Ibiebele I (2011). Stillbirths: Where? When? Why? How to make the data count?. Lancet.

[25] Pagel C, Prost A, Lewycka S, Das S, Colbourn T, Mahapatra R (2011). Intracluster correlation coefficients and coefficients of variation for perinatal outcomes from five cluster-randomised controlled trials in low and middle-income countries: results and methodological implications. Trials..

[26] Hemming K, Girling AJ, Sitch AJ, Marsh J, Lilford RJ (2011). Sample size calculations for cluster randomised controlled trials with a fixed number of clusters. BMC Med Res Methodol.

[27] Boen RJ. P values versus mean and standard errors in reporting data. Letters J JAMA. 1969;208. 10.1001/jama.1969.03160030109025 5818537

[28] Du Prel J-B, Hommel G, Röhrig B, Blettner M (2009). Confidence interval or p-value?. DtschArztebl Int.

[29] Pearson ES. The Use of Confidence or Fiducial Limits. 1988;26:404–13.

[30] R Core Team. R: a language and environment for statistical computing. Vienna: Foundation for Statistical Computing, 2014. Available at http://www.R-project.org/. Accessed 11 Nov 2017.

[31] Sharma J, Kumari S, Dumka N, Singhal S, Correia B, Kannappa R, et al. Does the WHO Safe Childbirth Checklist improve the quality of care at childbirth in rural India? Evidence from a qualitative study in Rajasthan. (Manuscript under review).

[32] Bayou G, Berhan Y (2012). Perinatal mortality and associated risk factors: a case control study. Ethiopian J Health Sci.

[33] Ngoc NTN, Merialdi M, Abdel-Aleem H, Carroli G, Purwar M, Zavaleta N (2006). Causes of stillbirths and early neonatal deaths: data from 7993 pregnancies in six developing countries. Bull World Health Organ.

[34] Carlo WA, Goudar SS, Jehan I, Chomba E, Tshefu A, Garces A, Sailajanandan P, Althabe F, EM MC, Derman RJ, Goldenberg RL (2010). Newborn-care training and perinatal mortality in developing countries. N Engl J Med.

[35] Carlo WA, McClure EM, Chomba E, Chakraborty H, Hartwell T, Harris H (2010). Newborn care training of midwives and neonatal and perinatal mortality rates in a developing country. Pediatrics..

[36] Goudar SS, Derman RJ, Honnungar NV, Patil KP, Swamy MK, Moore J (2015). An intervention to enhance obstetric and newborn Care in India: a cluster randomized-trial. Matern Child Health J.

[37] Pasha O, McClure EM, Wright LL, Saleem S, Goudar SS, Chomba E (2013). A combined community- and facility-based approach to improve pregnancy outcomes in low-resource settings: a global network cluster randomized trial. BMC Med.

[38] Stanton C, Lawn JE, Rahman H, Wilczynska-Ketende K, Hill K (2006). Stillbirth rates: delivering estimates in 190 countries. Lancet.

[39] Cousens S, Blencowe H, Stanton C, Chou D, Ahmed S, Steinhardt L (2011). National, regional, and worldwide estimates of stillbirth rates in 2009 with trends since 1995: a systematic analysis. Lancet..

[40] Rajagopal VM, Betha K, Priya GS (2017). Classification of stillbirth by relative condition at death (re co De) at various trimesters of pregnancy: a rural tertiary teaching hospital based study. Int J Reprod Contracept Obstet Gynecol.

[41] Yagnik A, Gokhle AV (2016). Study of cases of still births at tertiary maternity care hospital (ReCoDe). Int J Med Res Health Sci.

[42] McClure EM, Saleem S, Goudhar SS, Moore JL, Garces A, Esamai F, et al. Stillbirth rates in low-middle income countries 2010–2013: a population-based, multi-country study from the Global Network. Reprod Health 2015; 12(2):S7. 10.1186/1742-4755-12-S2-S7 PMC4464024 26063292

[43] Dandona R, Kumar GA, Kumar A, Singh P, George S, et al. Identification of factors associated with stillbirth in the Indian state of Bihar using verbal autopsy: A population-based study. PLOS Med. 2017;14(8):e1002363. 10.1371/journal.pmed.1002363. Accessed 18 Dec 2018. 10.1371/journal.pmed.1002363 PMC5538635 28763449

[44] Maternal Health Division Ministry of Health and Family Welfare Government of India. Dakshata, Empowering providers for improved MNH care during institutional deliveries. Operational guidelines. 2015. Available at: http://nrhm.gov.in/images/pdf/programmes/maternal-health/guidelines/Dakshata-Operational_Guidelines.pdf . Accessed 5 Jan 2018.

